# Yoga versus health education for persistent fatigue in patients with post-COVID-19 syndrome: protocol for a multicentre randomised controlled trial

**DOI:** 10.1136/bmjopen-2025-107840

**Published:** 2025-10-23

**Authors:** Holger Cramer, Lisa Mörchen, Jan Vagedes, Jochen Klenk, Simon Jäger, Alina Schleinzer, Dennis Anheyer, Mirela Bilc, Marleen Schröter, Christian Kessler, Michael Jeitler

**Affiliations:** 1 Institute for General Practice and Interprofessional Care, University Hospital Tübingen, Tübingen, Germany; 2 Robert Bosch Center for Integrative Medicine and Health, Bosch Health Campus, Stuttgart, Germany; 3 ARCIM Institute, Filderklinik, Filderstadt, Germany; 4 Department of Neonatology, University Hospital Tübingen, Tübingen, Germany; 5 Institute of Epidemiology and Medical Biometry, Ulm University, Ulm, Germany; 6 Department of Clinical Gerontology, Robert-Bosch Hospital, Stuttgart, Germany; 7 IB University of Health and Social Sciences, Study Center Stuttgart, Stuttgart, Germany; 8 Insitute of Clinical Pharmacology, Klinikum Nürnberg and Paracelsus Medical University, Nürnberg, Germany; 9 Department of Psychology and Psychotherapy, Chair of Research Methodology and Statistics in Psychology, University Witten/Herdecke, Witten, Germany; 10 Charité Competence Center for Traditional and Integrative Medicine (CCCTIM), Charite - Universitatsmedizin Berlin, Corporate Member of Freie Universität Berlin and Humboldt-Universität zu Berlin, Berlin, Germany; 11 Institute of Social Medicine, Epidemiology and Health Economics, Charité - Universitätsmedizin Berlin, Corporate Member of Freie Universität Berlin and Humboldt-Universität zu Berlin, Berlin, Germany; 12 Department of Internal and Nature-Based Therapies, Immanuel Hospital Berlin, Berlin, Germany

**Keywords:** post-acute COVID-19 syndrome, fatigue, clinical protocols

## Abstract

**Introduction:**

Post-COVID-19 syndrome, defined by persistent symptoms lasting beyond 12 weeks of a SARS-CoV-2 infection, affects both severe and mild COVID-19 cases. Fatigue is the most common symptom, impacting 58% of patients. Other symptoms include mental symptoms, cardiovascular and respiratory issues and autonomic dysfunction. Chronic inflammation and immune dysregulation seem to be associated with post-COVID-19 fatigue. Despite its impact on healthcare and the economy, effective treatments are limited. Yoga and health education have been shown to be effective for fatigue in other related conditions. The aim of this study, therefore, is to investigate the efficacy, safety and cost-effectiveness of yoga and health education on post-COVID-19 persistent fatigue.

**Methods and analysis:**

A randomised controlled trial with 100 patients with persistent fatigue due to post-COVID-19 syndrome is being conducted at three study centres. Patients are randomised to two interventions, yoga and health education. Both interventions include 12 weeks of 90 min supervised group sessions and 60 min of home practice per week. The primary outcome measure is fatigue on the Chalder Fatigue Scale 12 weeks after randomisation. Secondary outcome measures include postexertional malaise (DePaul Symptom Questionnaire), health-related quality of life (Short Form Health Survey-12 Item Version, EuroQol 5-Dimension 5-Level Questionnaire), anxiety, depression (Hospital Anxiety and Depression Scale), stress (Perceived Stress Scale), sleep quality (Pittsburgh Sleep Quality Index), hand grip strength, laboratory parameters and adverse events. Physical activity analysis over 7 days using a body-worn sensor and 24-hour heart rate variability using a 3-channel ECG recorder are assessed exploratively. All outcome measures will be assessed 12 and 24 weeks after randomisation. In addition, health economic analyses as well as mediator and moderator analyses including self-reported body awareness, self-efficacy, personality traits and treatment credibility/expectations will be conducted. Furthermore, qualitative interviews at week 12 will be carried out.

**Ethics and dissemination:**

The trial received ethical approval from the Ethics Committee of the University Hospital Tübingen (approval number: 775/2022BO2). Results will be disseminated via peer-reviewed open-access publications, scientific conferences and targeted communication to patient organisations, healthcare providers and the wider public.

**Trial registration number:**

NCT05890599.

**STRENGTHS AND LIMITATIONS OF THIS STUDY**

- This multicentre randomised controlled trial includes an active control group (health education), allowing for a more rigorous evaluation of yoga-specific effects beyond non-specific attention and group interaction.
- The study uses a comprehensive set of both subjective (eg, fatigue, quality of life) and objective (eg, hand grip strength, heart rate variability, activity sensors) outcome measures.
- The inclusion of health economic evaluation from a societal perspective provides valuable insights into the cost-effectiveness of interventions.
- The qualitative component enables in-depth exploration of participants’ subjective experiences, complementing the quantitative findings.
- Due to the nature of behavioural interventions, blinding of participants is not feasible, which may introduce expectancy or reporting bias.

## Background

 Patient data from around the world show that the health of patients suffering from COVID-19 can be impaired by late effects months after acute infection.^1^,^5^ The National Institute for Health and Care Excellence (NICE) guideline and the German guideline on long covid/post-COVID-19 characterise post-COVID-19 syndrome as a symptom cluster that occurs within 12 weeks of SARS-CoV-2 infection, lasts for at least 8 weeks and cannot be explained by another diagnosis.^6 7^ Symptoms can persist after an acute COVID-19-19 infection or reappear after initial recovery.^6 8^

The prevalence of post-COVID-19 syndrome among patients with a severe course of the disease and a resulting hospitalisation is reported to be up to 80%.^9^ However, patients with supposedly mild courses can also be affected.^9 10^ Fatigue is the most frequent and disabling symptom, reported by around one-third of patients overall and by 58% of those with post-COVID-19 syndrome.^9^

In addition to fatigue, respiratory and cardiovascular complaints are common. Organ damage has mainly been described in patients with ‘postintensive care syndrome’.^11^,^13^ Cardiovascular manifestations include tachycardia, palpitations and postural orthostatic tachycardia syndrome.^9 14 15^ Alterations in cardiac autonomic function, particularly reduced heart rate variability (HRV), have been reported.^16^,^18^ Such dysautonomia is also known from cancer-related and other postviral fatigue syndromes.^18 19^

Fatigue is a severe exhaustion on a somatic, functional, cognitive and/or psychological level that is disproportionate to the previous exertion and does not improve sufficiently with rest.^20^ It has been observed after various viral infections and partly overlaps with myalgic encephalomyelitis/chronic fatigue syndrome (ME/CFS).^20^,^22^ Proposed mechanisms include low-grade inflammation, autoimmunity, virus persistence, autonomic dysfunction and microthrombi.^23^,^27^ Postexertional malaise (PEM), an acute exacerbation of symptoms after exertion, is also frequently reported.^728^,^30^

Post-COVID-19 fatigue has substantial consequences for quality of life, social participation and work capacity, with high socioeconomic costs.^31^,^34^

The treatment of fatigue has so far mainly been investigated in the context of cancer-related fatigue.^35^ Among non-pharmacological approaches, physical activity is considered most effective.^35^,^38^ However, for patients with post-COVID-19, exercise tolerance is often limited, and standard programmes may trigger PEM.^7 39^ The Association of the Scientific Medical Societies in Germany (German: Arbeitsgemeinschaft der Wissenschaftlichen Medizinischen Fachgesellschaften, AWMF) S1-long covid/post-COVID-19 guideline recommends exercise therapy with a biopsychosocial focus and the core components of endurance and strength training supervised by trained personnel as part of rehabilitation in patients with post-COVID-19 syndrome.^7^ One lower-intensity form of physical activity could be yoga.

Yoga combines physical postures, breathing techniques and meditative elements.^40^ In recent decades, it has been increasingly applied as a therapeutic intervention. Objectives include strengthening the body, enhancing body awareness and promoting stress regulation and self-efficacy.^40^ Practices can be adapted to individual functional levels, enabling fatigue-paced group sessions.

Yoga research in clinical populations—including cancer, depression, cardiac arrhythmias, chronic back pain and type 2 diabetes—demonstrates preventive and therapeutic benefits, reducing stress, anxiety, depression, sleep disorders, pain and fatigue, while improving quality of life, mood, sleep and inflammatory processes.^35^,^3741^

Furthermore, beneficial effects on cardiovascular regulation and HRV have been reported.^55 56^ Yoga is generally safe and associated with good adherence.^57^

Taken together, yoga may address both the physical and psychological symptom clusters of post-COVID-19 syndrome and thus represent a promising therapeutic approach.^58 59^

### Objectives

The aim of this study is to investigate whether yoga is a useful therapeutic approach for post-COVID-19 persistent fatigue. The primary objective of this intervention study is to evaluate the short-term effect of yoga in addition to routine care on fatigue in patients with post-COVID-19 compared with health education in addition to routine care. Secondary objectives include the evaluation of medium-term effect on fatigue as well as short-term and medium-term effect of a yoga intervention in addition to routine care on health-related quality of life, mental health symptoms, performance limitations, sleep quality, stress and inflammation in patients with post-COVID-19 syndrome compared with health education in addition to routine care. The safety of the interventions is being investigated through close monitoring of adverse events (AEs). The study further uses cost-effectiveness and cost-utility analysis to investigate how cost-effective the yoga group is compared with the health education group. Furthermore, qualitative interviews will be conducted following the interventions to capture the subjective experiences of the patients.

## Methods

### Study design

The study is designed as a multicentre, single-blind randomised controlled trial with two study arms. The outcome assessor is blinded to group allocation. The study protocol is reported according to the Standard Protocol Items: Recommendations for Interventional Trials guideline.^60^

**Figure 1:**
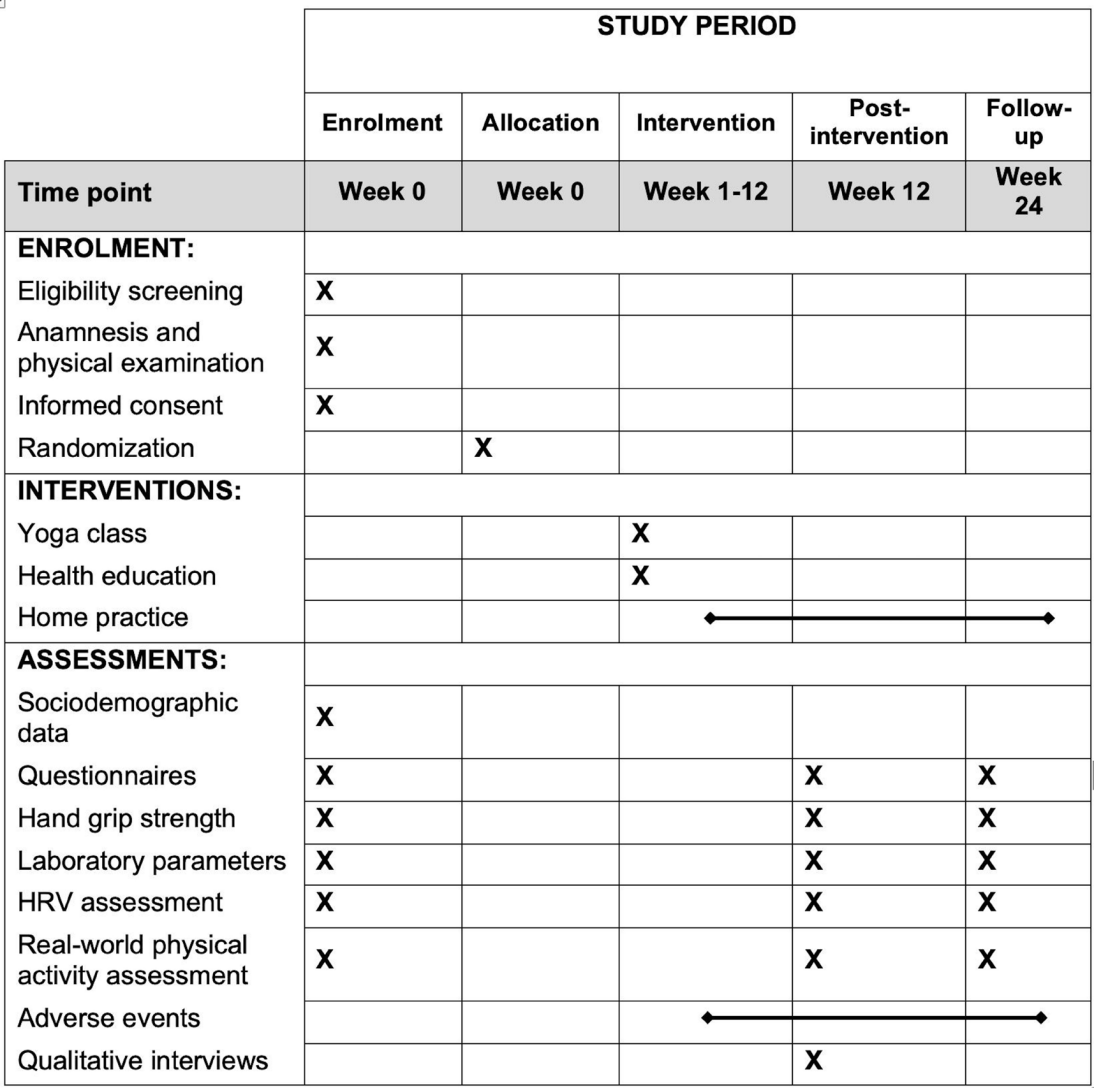
SPIRIT-recommended content for the schedule of enrolment, interventions and assessments. HRV, heart rate variability; SPIRIT, Standard Protocol Items: Recommendations for Interventional Trials.

The study centres are the Institute of General Practice & Interprofessional Care at the University Hospital Tübingen, the Robert Bosch Center for Integrative Medicine and Health at Bosch Health Campus Stuttgart and the Charité Integrative Medicine Outpatient Department at Immanuel Hospital Berlin-Wannsee. A period of 12 weeks after randomisation is scheduled per participant until the primary outcome measure is collected (figure 1). Sustainability will be assessed as a follow-up after 6 months.

###  Ethical approval, trial registration and trial status

The research ethics committee of the University Hospital Tübingen (approval number: 775/2022BO2) granted ethical approval prior to participant recruitment. The trial was initially registered with ClinicalTrials.gov (NCT05890599) on 2 June 2023, based on protocol V.1.0, prior to participant recruitment. A first amendment dated 6 June 2023 added the following to the measurements: the questionnaires EuroQol 5-Dimension 5-Level Questionnaire (EQ-5D-5L), Short Form Health Survey-12 Item Version (SF-12), PEM-DePaul Symptom Questionnaire (DSQ), Body Awareness Questionnaire (BAQ) and Big Five Inventory (BFI-­10); the laboratory parameters D-dimers, lactate dehydrogenase (LDH), leucocytes and lymphocytes as well as assessments of hand grip strength (HGS), real-world physical activity and HRV. A second amendment dated 14 June 2025 added the qualitative aspects to the study. All amendments and updated protocol versions were communicated to ClinicalTrials.gov to ensure transparency. This manuscript refers to V.1.3 of the protocol (17 January 2025), which incorporates these updates. Participant recruitment started in 10 June 2023 and is planned to be completed by September 2025. The study is expected to be completed by March 2026.

### Study population 

Patients with persistent fatigue as a result of post-COVID-19 syndrome are included in the study population. Based on the German long covid/post-COVID-19 S1 guideline (as of 5 March 2023), three categories are used to diagnose post-COVID-19 syndrome: (1) symptoms that persist after the acute COVID-19 infection or its treatment, (2) new symptoms that occur after the end of the acute phase but can be understood as a consequence of SARS-CoV-2 infection and (3) worsening of a pre-existing condition as a result of SARS-CoV-2 infection.^7^

The definition given in the widely used NICE guidelines states that post-COVID-19 syndrome includes ‘signs and symptoms that develop during or after an infection consistent with COVID-19, continue for >12 weeks and are not explained by an alternative diagnosis. It usually presents with clusters of symptoms, often overlapping, which can fluctuate and change over time and can affect any system in the body’.^6^ To differentiate from patients with long covid syndrome, patients are only included if symptoms persist for > 12 weeks.

### Inclusion and exclusion criteria

#### Inclusion criteria

- Confirmed COVID-19 infection, diagnosed by PCR or serology (SARS-CoV-2 IgG/IgA).
- Subsequent symptoms (as described in the NICE guidelines) lasting >12 weeks
- Self-assessment that the fatigue is a consequence of COVID-19 disease.
- Presence of persistent fatigue, operationalised by scores ≥4 on the binomial Chalder Fatigue Scale (CFQ).^61^
- Age 18–65 years.

#### Exclusion criteria

- Chronic disease or regular use of medication that is associated with fatigue (eg, untreated hypothyroidism, diabetes, autoimmune diseases, anaemia defined as haematocrit <24, chronic fatigue syndrome due to causes other than COVID-19, endocrinopathies, cancer, heart failure).
- Indications that fatigue is primarily caused by another medical or psychiatric condition other than post-COVID-19 (eg, major depression, insomnia, sleep apnoea).
- Indications that fatigue is primarily caused by other factors (eg, shift work, recent change in activity or schedule).
- Physical limitations or conditions that preclude participation in the yoga intervention (eg, severe neck injuries, unstable joints, severe pre-existing cardiovascular disease, organ failure (kidney, liver, etc), acute febrile infection, severe neurological diseases).
- Pregnancy or breastfeeding.
- Simultaneous participation in other clinical trials.
- Simultaneous participation in other yoga interventions.

### Recruitment 

Participant recruitment takes place via notices and leaflets at the University Hospital Tübingen, the Bosch Health Campus Stuttgart, Charité - Universitätsmedizin Berlin and in post-COVID-19 focused practices and self-help groups in the regions, as well as via calls in the local media and in social networks (eg, Instagram). Potential participants are screened by telephone. If the eligibility criteria are preliminarily met, the inclusion and exclusion criteria will be checked by an experienced researcher at the respective study centre and an anamnesis and physical examination will be carried out by an experienced study physician to further assess eligibility as well as the feasibility and safety of the study interventions and measurements for the specific participant. If the inclusion criteria are met, there are no exclusion criteria, and the interventions and measurements are deemed feasible and safe, participants can be included in the study. All prospective participants first receive detailed verbal and written information on the study and have the opportunity to ask questions. A written informed consent declaration is required before participation in the study.

### Randomisation 

After completion of the measurements at week 0, eligible participants will be assigned to one of two study arms, the yoga group or the health education group, via block randomisation (stratified by gender and study centre) with randomly varying block length. The randomisation sequence was created by an external scientist who has no participant contact during the entire study using R software (V.4.3.1). The randomisation list will be kept password-protected and only the scientist creating the list has access to it. Allocation concealment is ensured by central randomisation via Research Electronic Data Capture (REDCap) software. Once a participant has been randomised by the study physician, their data can no longer be changed in REDCap. Neither the participants nor the study physicians nor the study coordinators have insight into the randomisation sequence.

### Blinding

Blinding of participants and intervention providers is not feasible due to the nature of behavioural interventions. However, outcome assessment is blinded wherever possible. Performance-based measures (eg, HGS, physical activity sensors, HRV) are conducted by study staff who are unaware of participants’ group allocation. Laboratory analyses are carried out externally without access to allocation data. For the qualitative interviews, complete blinding cannot be ensured, as participants may refer to their intervention experiences. To reduce potential bias, interviews are conducted by researchers not involved in intervention delivery, and participants are not actively asked to reveal their group allocation. These procedures are implemented to minimise detection bias and to safeguard the validity of outcome assessment.

### Sample size calculation

The study is powered to detect a minimal clinically relevant effect of 3.3 points on the CFQ.^61^ Based on an SD of 4.4 in a sample of patients with post-COVID-19 syndrome and fatigue diagnosed by CFQ,^62^ the estimated effect size is d=0.75. Assuming this effect, a two-tailed independent t-test with a significance level of α=0.05 requires a total of 78 participants to detect a group difference with a power of 90%. In order to avoid a possible loss of power due to a dropout rate of up to 20%,^63^ it is planned to include 100 participants in the study and assign them to the two study groups in a 1:1 ratio. Sample size calculation was performed using the ‘pwr’ package in R V.4.3.1.^64^

### Interventions 

**Yoga**

The content of the yoga treatment was developed on the basis of systematic literature research on previous yoga interventions, particularly for patients with cancer and patients with ME/CFS, as well as the experience of an interdisciplinary team with several years of experience in yoga therapy, integrative medicine and clinical practice. The yoga treatment is carried out by certified and experienced yoga teachers once a week in consistent groups of a maximum of 11 participants on site and covers a total period of 12 weeks. All yoga instructors hold a minimum 500 hours yoga teacher certification from a recognised yoga alliance and have additional experience in yoga therapy or clinical applications. Prior to the start of the trial, instructors participated in a standardised preparatory training conducted by the study team, covering post-COVID-19 symptomatology, PEM and pacing, as well as the study-specific manual. Adherence to the intervention manual is supported by the detailed written curricula, standardised video material and two supervision meetings (one during and one after the intervention) with the study centre.

The intervention includes the three main yogic elements: yoga postures (āsāna), breathing techniques (prānāyāma) and meditation (dhyāna). It lays an emphasis on postures and breathing techniques considered effective for fatigue with a focus on laying positions (as in isometric yoga), seated poses (practised on a chair), side bends, passive inversion postures and backbends (ie, supported spinal extensions) as well as targeted breathing exercises to strengthen the respiratory muscles and increase the lung volume by mobilising the diaphragm.

The intervention duration of 150 min per week is based on previous studies on yoga interventions specifically for fatigue patients and on recommendations such as those of WHO, which recommend at least 150 min of moderate-intensity aerobic physical activity per week spread over at least 3 days per week for adults, including people with chronic illnesses.^65^ The German guideline on long covid/post-COVID-19 recommends a training duration of 40–60 min, 3–6 times per week.^7^ However, previous experience with this patient population suggested that it is difficult for patients to travel to the study site several times a week to attend yoga classes, while regular attendance is of great importance for the success of the treatment.^37 54^ It was therefore decided that the group would only meet once a week on site, for a total duration of 90 min including a maximum of 40–60 min of āsāna (posture) practice. Each session starts with a 15 min group check-in time, where participants can share their weekly experiences, followed by 5–15 min of meditation (including Om-sounding) and bhāvana (setting an intention for the class) and a 5–10 min breathing exercise. Each session is concluded by 9–10 min of rest in shavāsāna and 4 min group closing. To conclude the total physical activity time of 150 min a week, participants have access to yoga videos specially developed for the study and based on the on-site programme. The online material consists of 19 yoga videos of varying length and intensity (duration 0:48 to 47:04 min) and is sectioned by ‘energy level’ needed to practise them. This ensures that participants receive the recommended amount of yoga, but that the yoga practice can be better integrated into everyday life, thereby promoting compliance. Participants are encouraged to engage in pacing-orientated, home-based yoga practice for approximately 60 min per week. The duration of home practice will be recorded through self-reported weekly practice times.

### Health education

Health education is an established therapy for the treatment of cancer-related fatigue, for example, in patients with breast cancer, and is delivered by experienced therapists.^66^ The health education courses are held once a week for 90 min over 12 weeks on site by healthcare professionals. All educators are experienced clinicians (eg, physicians, physician assistants, physiotherapists, nurses) and received a structured briefing on post-COVID-19 symptomatology, PEM and pacing as well as a detailed teaching manual before study initiation. The courses are didactic in nature and consist of lectures on topics of interest to patients with post-COVID-19 syndrome and fatigue, followed by questions and discussion sessions. The 12-week programme contains the following topics: (1) introduction, behaviour change and balance of health; (2) post-COVID-19 syndrome, fatigue and ME/CFS; (3) autonomic nerve system, relaxation and stress management; (4) movement and pacing with post-COVID-19 syndrome; (5) sleep and sleep hygiene; (6) internal communication and cognitive restructuring; (7) external communication; (8) nutrition with fatigue; (9) dealing with symptoms of anxiety, depression and post-traumatic stress syndrome; (10) social network; (11) everyday strategies and resilience and (12) review and relapse prevention. Each session starts with a 15 min group check-in time, where patients can share their weekly experiences and in-between lecture sections patients are engaged in group work or reflection practices. To ensure fidelity, all sessions follow a standardised manual and presentation slides, supplemented by two supervision meetings (one during and one after the intervention) with the study centre. The health education group, like the yoga group, will only meet once a week for 90 min. In addition, the aim is to consolidate and apply what has been learnt for 60 min per week at home. For this purpose, the participants receive the course material and eight health education videos (duration 7:54–14:04 min) specifically developed for patients with post-COVID-19 syndrome through an online platform.

Participants received no financial compensation. All participants may continue their routine care as prescribed, for example, by their general practitioner. Adherence to the interventions will be assessed.

## Outcome measures

### Primary outcome measures

#### Fatigue

In its standard version (range 0–33), the CFQ is used to assess the severity of fatigue within the last 4 weeks. It comprises 11 items in a 5-point Likert scale format (0–4 points), seven of which relate to the extent of physical exhaustion and four to the extent of mental exhaustion. In its binomial evaluation (range 0–11), it can be used to categorise patients with and without fatigue with a cut-off of ≥4 indicating fatigue.^65 67^ The CFQ demonstrates strong internal consistency with split-half reliabilities of 0.85 and Cronbach’s α values ranging from 0.86 to 0.92.^68^

### Secondary outcome measure

#### Postexertional malaise

The DSQ is a standardised instrument for ME/CFS diagnosis.^69 70^ Its validated PEM subscale has been used since 2020 to assess PEM frequency and PEM severity in patients with post-COVID-19 syndrome.^22 71^ The PEM subscale comprises five items on frequency, five on severity and five on duration and classification.^71^

#### Health-related quality of life

The SF-12 is the most commonly used instrument in clinical studies to measure generic health-related quality of life. The SF-12, a validated 12-item short form of the SF, shows no significant difference to its longer versions in the assessment of the physical and mental component score. For this reason, the SF-12 is used to reduce the burden on fatigue patients when completing the questionnaires.^72^,^74^

#### Mental health

The Hospital Anxiety and Depression Scale comprises 14 items, including seven items on anxiety and seven items on depression in the past week in participants with physical illnesses and physical complaints. More precisely, it measures the participants’ self-assessed level of anxious and depressive symptoms in relation to the past week.^75^ Participants’ current perception of stress is assessed using the Perceived Stress Scale-10 Item Version.^76^ This questionnaire measures the participants’ current subjective perception of stress using 10 items in the four subscales of worry, tension, pleasure and demands.^77^

#### Sleep quality

The Pittsburgh Sleep Quality Index uses 24 items, 17 for self-assessment and seven for external assessment, to measure the quality of sleep over the past 4 weeks. It is used in clinical settings to monitor patients with sleep disorders and to screen for sleep disorders in epidemiological studies.^78^ In this study, only the 17 self-assessment items are collected.

#### Hand grip strength

HGS is measured in kilograms using a digital hand dynamometer (CAMRY, model: SCACAM-EH101) as an objective parameter of muscular fatigue. The participants complete two separate rounds of pulling 10 times for 3 s with maximum force, the second round in a distance of 1 hour to the first, spaced by filling out questionnaires as a muscular recovery break of 60 min. The participants are supervised by a study nurse or study physician and not allowed to see their HGS. Within the 3 s of pulling time, the CAMRY dynamometer measures the maximum pulling strength. The measurement with the highest value out of the 10 repetitions is recorded as maximum strength of the round.

### Laboratory parameters

Leucocytes, lymphocytes, D-dimers, C reactive protein, interleukin-6 and LDH are determined from blood samples as parameters for the inflammatory reaction, pro-inflammatory cytokine activity and cell damage.^26^

### Real-world physical activity assessment

The EC-certified sensor (Axivity AX6, Axivity, Newcastle, UK) used in this study includes an accelerometer and a gyroscope. It records the duration of activity and is able to objectively quantify different activity domains, which are related to the physical fatigue and PEM frequency of patients. The sensor is placed centrally on the lower back, close to L5, and is attached with a skin-friendly and waterproof plaster. The sensor is worn continuously for 7 days over 24 hours and can be worn during all daily activities, including showering. A software platform is used to process the raw sensor data (McRoberts, The Hague, The Netherlands).

The following activity parameters are derived for each day of the study using validated algorithms:

- Cumulative activity time (total and specific activities, eg, walking, shuffling, cycling, standing and static).
- Number of transitions (eg, sit-to-stand transfers).
- Number of periods (eg, number of walking bouts total and with specific durations, eg, >10 s).
- Number of steps (average, minimum, maximum, range).
- Walking speed (average, minimum, maximum, range).
- Walking distance (total, maximum within a bout).
- Duration of activity in specific metabolic equivalent (MET) ranges (eg, total duration of periods below three METs).

The following non-activity parameters are also derived for each day of the study:

- Cumulative non-activity time (total and specific non-activities: lying and sitting).
- Cumulative lying during daytime and nighttime.
- Movement during lying time (eg, duration, number of periods).

### Heart rate variability

The 24-hour measurement of HRV is used to determine the functionality of the participant’s autonomic nervous system. A 3-channel ECG recorder (SRA/Multi-ECG recorder, SR-Medizinelektronik, Stuttgart, Germany) is used. Several HRV time-domain and HRV frequency-domain measures will be evaluated and analysed for the total day and night (sleeping time) period as well as for 5 min intervals within these two periods (table 1).^79^

**Table 1:** HRV measures

HRV time-domain measures		
**Parameter**	**Unit**	**Description**
SDNN	ms	SD of NN intervals
RMSSD	ms	Root mean square of successive RR interval differences
pNN50	%	Percentage of successive RR intervals that differ by >50 ms
SDANN	ms	SD of the average NN intervals for each 5 min segment of a 24-hour HRV recording
SDNNI	ms	Mean of the SD of all the NN intervals for each 5 min segment of a 24-hour HRV recording
**HRV frequency-domain measures**		
**Parameter**	**Unit**	**Description**
VLF power	ms^2^	Absolute power of the very-low-frequency band (0.0033–0.04 Hz)
LF power	ms^2^	Absolute power of the low-frequency band (0.04–0.15 Hz)
LF power	%	Relative power of the low-frequency band (0.04–0.15 Hz)
HF power	ms^2^	Absolute power of the high-frequency band (0.15–0.4 Hz)
HF power	%	Relative power of the high-frequency band (0.15–0.4 Hz)
LF/HF	%	Ratio of LF-to-HF power

HRV, heart rate variability.

### Health economics

**Figure 2:**
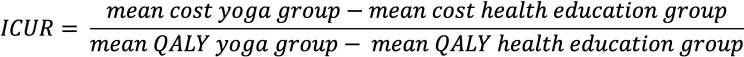
Incremental cost-utility ratio (ICUR).

The societal cost-effectiveness and cost-utility analyses will be conducted to compare the costs as well as the effect and utility of the yoga and health education group. Accordingly, all relevant costs will be included in the economic evaluation. The cost-effectiveness analysis will use the CFQ score as the outcome measure, while the cost-utility analysis will use quality-adjusted life years (QALYs).^80^ The incremental cost-effectiveness ratio is calculated based on the incremental costs and incremental CFQ scores. Similarly, the incremental cost-utility ratio (ICUR) is calculated based on the incremental costs and incremental QALYs (figure 2).^81^

### Measuring costs

The end of the intervention, that is, 12 weeks after randomisation, and 24 weeks after randomisation are the time points for cost collection. In order to capture all relevant costs for an economic analysis, the social perspective is chosen. Thus, intervention costs, direct medical and non-medical costs as well as productivity losses are considered. All costs are expressed in euros (€).

Direct medical and non-medical costs are collected using a participant-completed questionnaire. The questionnaire is a modified version of the Cost for Patient Questionnaire,^82^ designed to collect all information on healthcare costs. Costs are assessed according to their standard price or according to the reimbursement system in Germany. The direct costs of the interventions are collected during the intervention, recorded in the study documents and valued at their actual cost. Indirect costs are expressed in terms of productivity losses and are recorded in the participant questionnaire. Productivity losses for employed participants are estimated by multiplying the productivity hours lost by the average national employment cost. Productivity losses are included in the sensitivity analysis.

### Measuring utility

Utility of the intervention is measured by calculating QALYs using the EQ-5D-5L questionnaire at baseline, after 12 weeks and after 24 weeks.

The EQ-5D-5L is a preference-based generic instrument for measuring health-related quality of life.^83^ It consists of a self-assessment section and a EuroQol visual analogue scale (VAS). The self-assessment comprises a five-level classification system for five dimensions of quality of life. On the VAS, participants rate their health-related quality of life on a scale from 0 mm to 100 mm. The main advantage of the EQ-5D-5L over the SF-12 is that it provides a preference-based quality of life index score across all dimensions, which is necessary for calculating QALYs and conducting a cost-utility analysis. For this reason, it is used in addition to the SF-12.^84 85^

QALYs are calculated by recording utilities at each follow-up point and estimating the area under the curve based on social preference weights provided by the EuroQol group. To avoid bias, we will adjust for differences in baseline EQ-5D-5L scores.

### Potential mediating variables

#### Body awareness

BAQ assesses participants’ subjective body awareness in four subdimensions: note responses or changes in body processes, predict bodily reaction, sleep-wake cycle and onset of illness. The validated single-factor German version consists of 17 questions on a 7-point Likert scale from 1 (not at all true about me) to 7 (very true about me). The values of the individual items are added together to form a total score.^86 87^

#### Self-efficacy

The Arthritis Self-Efficacy Scale (ASES) was originally developed as part of the Stanford Arthritis Self-Management Study to assess self-efficacy in patients with arthritis and has since been used to assess this construct in many clinical pictures. A short version (ASES-8/ASES-D) has been validated. This version measures the perceived ability to deal with symptoms such as pain or fatigue on a scale ranging from 1 to 10.^88^

### Potential moderating variables and covariates

#### Personality traits

The Big Five Inventory-10 is a questionnaire for the efficient assessment of the five dimensions of personality according to the five-factor model (Big Five model). It consists of 10 items. Two items each (one positive and one negative) are used for each of the five main dimensions of personality (extraversion, agreeableness, conscientiousness, neuroticism and openness to experience). The answers are given on a 5-point rating scale ranging from 1 (strongly disagree) to 5 (strongly agree). The values on the two items are aggregated into one scale value.^89^

#### Treatment credibility and expectations

The Credibility Expectancy Questionnaire is used to measure treatment credibility and expectations at the beginning of the study via six items using 9-point Likert scale.^90^

#### Safety management protocol 

AEs (containing all undesirable symptoms, illnesses, disorders or accidents), including PEM, are documented weekly by participants and reviewed by the study team every 2 weeks. Serious adverse events (SAEs) including events that are immediately life-threatening and/or result in serious health problems (in particular, require unplanned hospitalisation or prolongation of hospitalisation or result in serious disability or incapacity) must be reported immediately by direct telephone contact with the study site. PEM is systematically assessed for baseline and follow-ups using the validated PEM subscale of the DSQ and complemented by weekly symptom diaries. Predefined safety thresholds for modifying or discontinuing the intervention include (i) marked exacerbation of fatigue or other core symptoms persisting for >48 hours, (ii) deterioration in daily functioning that interferes with activities of daily living or (iii) occurrence of an SAE. Instructors and study physicians are authorised to adapt or interrupt the intervention in such cases. If participants are absent from two sessions due to health-related reasons, the site physician will discuss with them whether continued participation is considered safe and appropriate. No fixed cut-off score (eg, point value on Bell Score) was applied for exclusion. Instead, participants’ functional capacity was evaluated verbally during the physician-led informed consent visit, where risks and potential burdens of study participation were explained. This structured monitoring and safety management protocol is designed to minimise risk while ensuring the inclusion of a representative post-COVID-19 patient population.

### Qualitative interviews

After the end of the interventions, qualitative data will be collected from a subgroup (n=15–20) to evaluate the interventions from the participants‘ perspective. This will be done through semi-structured, guideline-based interviews (lasting approximately 20–30 min) with narrative components. The areas of interest include subjective experiences with the interventions, the perception of the illness and its impacts. Participants will be selected equally from both groups based on predefined criteria (eg, gender, adherence to the therapy programme). The interviews will be transcribed using software (noScribe) and analysed using qualitative content analysis according to Kuckartz in MaxQDA software.

### Data management

All data were collected and managed using the REDCap system. The REDCap database was programmed to ensure data quality through built-in validation rules and logic checks. Comprehensive strategies will be implemented to promote participant retention and ensure complete follow-up throughout the study. All reasonable efforts are made to maintain participant engagement, including regular communication, flexible scheduling and reminders. In cases where participants discontinue or deviate from the intervention protocol, relevant outcome data will continue to be collected as specified in the study protocol, whenever possible. These measures are intended to maximise data completeness and uphold the integrity of the study findings.

### Statistical analysis

All analyses are based on the intention-to-treat population, that is, all participants included in the study are analysed in the group to which they were originally assigned, regardless of the treatment they actually received. Missing values are handled using multiple imputation. The multiple imputation procedure will be conducted using R (V.4.3.1), employing the ‘missForest’ package for handling missing data.^91^ The multiple imputation procedure using the ‘missForest’ package involves handling missing values by employing a non-parametric approach. This package uses a Random Forest algorithm to predict missing values based on the observed data, accommodating both numerical and categorical variables. Through iterative variable iterations, missing values are imputed by leveraging predictions from the remaining variables within the dataset. In addition, a per-protocol analysis is conducted as a sensitivity analysis. The per-protocol population is defined as all participants who provide data at the respective time point and participate in at least 8 of the 12 intervention appointments.

The primary outcome measure is analysed confirmatory using a univariate analysis of covariance, in which the primary outcome measure is modelled as a function of group allocation (fixed factor), study centre (binary covariate), treatment credibility and expectation (linear covariates) and the respective baseline values (linear covariate). Within this model, the adjusted group difference and its 95% CI are estimated and tested for superiority of the intervention over the control group using a two-sided t-test at the α=0.05 level. The secondary end points are analysed exploratively (without correction for multiple testing) using the same models as the primary end point. To estimate the clinical significance of the results, change scores and effect sizes (Hedges’ g) are calculated for between-group comparisons.

HRV parameters within groups will be compared at two different time points using a Wilcoxon signed rank test. The results will be presented as medians and IQRs, Hodges-Lehmann estimator (HLE) of the differences, 95% CI and effect sizes r. A comparison between groups will be undertaken by examining the HLE, the 95% CI and the effect sizes r simultaneously.

All analyses will be conducted using R V.4.3.1, with the ‘psych’‘ package used for psychometric assessments.^92^

### Health economic analysis

As the analysis does not cover a period of >1 year, it is not necessary to discount the costs and QALYs incurred. Descriptive analyses will be conducted to determine the costs incurred in the yoga and health education group. Regression models will be used to identify possible predictors of cost or outcome differences. Due to the assumptions made and the estimated costs, there is a degree of uncertainty in the economic analyses. Thus, sensitivity analyses are performed to determine the robustness of the estimates to various assumptions.^93^ In addition, means and 95% CIs are estimated by bootstrapping using the observed distributions of the cost data in the analysed study.^94 95^ To summarise the uncertainty in the cost-effectiveness and cost-utility estimates, the cost-effectiveness acceptability curve and the cost-utility acceptability curve are calculated.^96^ The curves illustrate the probability that the cost-effectiveness or cost-utility ratio of the yoga intervention falls below a specified willingness-to-pay threshold.

### Mediator/Moderator analyses

To examine the potential mechanisms underlying the relationship between the independent and dependent variables, a mediation analysis will be conducted using the PROCESS macro for R.^97^ In line with theoretical considerations, body awareness and self-efficacy have been specified as potential mediators. The mediation model will test whether the effect of the independent variable on the dependent variable is mediated by a hypothesised mediator variable. Bootstrapping with 5000 resamples will be employed to estimate the indirect effect and its 95% CI.

To investigate potential moderation effects, a moderator analysis will be conducted using the PROCESS macro for R.^97^ The moderator analysis will focus on personality traits and treatment credibility and expectations as pre-specified moderators, examining whether the relationship between the independent and dependent variables is contingent on these factors. Interaction terms between the independent variable and the moderator will be included in the regression model to test for moderation effects. Bootstrapping with 5000 resamples will be employed to estimate the conditional effects of the independent variable on the dependent variable at different levels of the moderator.

### Ethics and dissemination

Ethical approval for the YoFaPoCo trial was granted by the Research Ethics Committee of the University Hospital Tübingen (approval number: 775/2022BO2). All participants receive detailed oral and written information about the study and provided written informed consent prior to participation. A model participant consent form is provided in the online supplemental file. Data collection and handling follow the General Data Protection Regulation. Direct identifiers (eg, name, contact details) are accessible only to authorised study staff involved in recruitment and follow-up; pseudonymisation and principles of data separation are applied to minimise re-identification risk. Data are stored on secure REDCap servers of University Hospital Tübingen with restricted access for named investigators only. Pseudonymised trial data will be retained for 10 years in accordance with institutional and legal requirements and may be shared with collaborating researchers on reasonable request.

The results will be disseminated through peer-reviewed open-access publications and presentations at national and international scientific conferences. In addition, results will be communicated to patient organisations, post-COVID-19 networks and healthcare providers. Lay dissemination will include press releases and social media updates to ensure that findings reach patients, caregivers and the wider public.

## Discussion

The present study protocol addresses a critical gap in the management of post-COVID-19 syndrome, with a particular focus on persistent fatigue, by evaluating yoga as a potentially effective, safe and cost-efficient intervention in comparison with health education. Recent research has highlighted the multidimensional nature of post-COVID-19 symptomatology, which frequently includes fatigue, reduced pulmonary function, cognitive impairment and psychological challenges such as anxiety and depression.^13^,^5^ The absence of universally effective treatment options for these enduring symptoms underscores the urgent need for innovative, accessible and patient-centred therapeutic strategies.

Emerging evidence suggests that yoga may provide benefits highly relevant to the treatment of post-COVID-19 syndrome. Preliminary studies indicate that yoga interventions can lead to significant improvements in fatigue, quality of life and lung function among individuals recovering from COVID-19.^98 99^ These effects are thought to be mediated by a combination of physical, physiological and psychological mechanisms. Yoga offers a form of physical activity that is both gentle and adaptable, allowing it to be tailored to individual energy levels and thereby minimising the risk of PEM while potentially enhancing adherence compared with more intensive exercise regimens.^100^ In addition, yoga appears to exert modulatory effects on the autonomic nervous system and may reduce inflammatory markers—both of which are implicated in post-COVID-19 fatigue and dysautonomia.^100^ Furthermore, the mind-body components of yoga, including breathwork and meditation, may support emotional regulation and reduce psychological distress. These aspects are particularly relevant given the high prevalence of anxiety, depression and stress among individuals with post-COVID-19 symptoms, all of which are known to exacerbate fatigue.^100^

This study is characterised by several methodological strengths. By comparing yoga with an active control condition—health education—it accounts for non-specific effects such as attention, social support and group interaction, thereby enabling a more accurate assessment of yoga’s specific therapeutic impact. The study also adopts a comprehensive assessment strategy that includes both subjective outcomes, such as self-reported fatigue and quality of life, and objective measures, such as HGS, HRV and laboratory parameters. Additionally, health economic analyses and qualitative interviews provide a multidimensional evaluation of intervention outcomes. Importantly, the economic evaluation takes a societal perspective, incorporating both direct and indirect costs, thereby offering valuable insights for health policy and resource allocation.

Nonetheless, several limitations should be acknowledged. As with many clinical trials, the generalisability of the findings may be limited, as the sample may not fully represent the broader post-COVID-19 population, particularly individuals with severe comorbidities or differing sociodemographic characteristics. While self-reported measures such as fatigue and quality of life are essential for capturing patient perspectives, they are subject to reporting bias. This limitation is partially mitigated by the inclusion of objective outcome measures. The follow-up period of 24 weeks allows for the evaluation of medium-term effects, but long-term outcomes remain to be determined. Additionally, participant blinding is not feasible in behavioural interventions such as yoga, which may introduce expectancy effects and influence self-reported outcomes.

### Implications for practice and research

If the study demonstrates favourable outcomes, yoga could be recommended as a low-risk, scalable intervention for the management of persistent fatigue and related symptoms in individuals with post-COVID-19 syndrome. The findings may support the integration of mind-body therapies into rehabilitation protocols and inform future clinical guidelines. From a health systems perspective, the economic analysis included in this study could justify the broader implementation of such interventions.

Future research should explore the long-term sustainability of yoga’s effects, identify optimal delivery formats (eg, online vs in-person) and assess its applicability across diverse patient populations and healthcare settings.^99^ Larger trials with extended follow-up periods will be necessary to confirm and expand on these findings.

## Conclusion

This protocol makes a meaningful contribution to the evolving field of post-COVID-19 care. By rigorously evaluating the effects of yoga in comparison with an active control group, the study aims to clarify the potential role of integrative therapies in addressing the complex and multifaceted needs of individuals affected by post-COVID-19 syndrome.

## Supplementary material

10.1136/bmjopen-2025-107840 online supplemental file 1

## Data Availability

No data are available.
