# Supplementary material for: Yoga versus health education for persistent fatigue in patients with post-COVID-19 syndrome: protocol for a multicentre randomised controlled trial
Source: BMJ Open. 2025 Oct 23;15(10):e107840. doi: 10.1136/bmjopen-2025-107840 (PMC12551510; doi:10.1136/bmjopen-2025-107840)
Supplement: online supplemental file 1 [file bmjopen-15-10-s001.docx]

**Institute for General Medicine and Interprofessional Care**

Chair for Research into Complementary Medicine

Osianderstr. 5

72076 Tübingen

Prof. Holger Cramer

holger.cramer@med.uni-tuebingen.de

Declaration of consent to participate in and data collection for the study:

Yoga versus health education for the treatment of persistent fatigue in patients with post-COVID syndrome (**YoFaPoCo**)

Patient: _______________________________________________________

(Last name, first name)

Date of birth: __ __ **.** __ __ **.** __ __ __ __

The subject was informed about the clinical study by

_____________________________________________________________

(investigator)

and covered the following points:

- Nature and purpose of the clinical trial
- Type and implementation of the yoga treatment and health education, including possible effects and side effects
- Type and implementation of the examination methods (questionnaires, blood sampling, qualitative interviews), including benefits and risks
- Information about the **non-existent** commuting accident insurance
- Information about the **non-existent** subject insurance
- Right to withdraw from the clinical study
- Information on data protection: Documentation, disclosure, and publication of subject data will be carried out in pseudonymized form
- We have been given a copy of the written subject information and the signed consent form

I agree to participate in the YoFaPoCo study and confirm that the informational discussion covered the points listed above. I am aware that I can withdraw my consent to participate in this clinical trial at any time without giving reasons and that this will not have any negative effects on my further treatment. All my questions about the study have been answered. **The withdrawal must be addressed to the study director, Prof. Dr. Holger Cramer (**Osianderstr. 5, 72076 Tübingen, Tel.: 0711/8101-2831, Email: holger.cramer@med.uni-tuebingen.de).

**____________________________ ________________________________**

Place, date Patient's signature

**___________________________ ________________________________**

Place, date Signature of the investigator

**Data protection**

**Declaration of consent to the handling of data collected in a study:**

**I declare that I consent to the collection and processing of data within the scope of the study and its encrypted (pseudonymized) transfer.**

**I agree that authorized persons may access my personal medical records for the purpose of verifying the data and release the attending physician from his or her medical confidentiality obligation in this regard.**

**I am aware that the results of this study will be published in medical journals, but in anonymized form, so that no direct reference to my person can be made.**

**I have been informed that I can request information about my stored data and the correction of incorrect data at any time.**

**I know that I can request at any time, for example when withdrawing from the study, that my data collected up to that point be deleted or anonymized immediately.**

**I declare that I have been adequately informed about the collection and processing of my data collected in this study and my rights.**

**I consent to the use of the data collected in this study in the manner described above. I release my treating physicians and the study team from their duty of confidentiality to the extent necessary for authorized persons to inspect the data.**

**I expressly agree that the study team may contact me even after the end of the study to ask whether I agree that the data collected as part of the study may also be used and further processed for specific future research projects of the clinic or institute.**

**Yes □ No □**

**____________________________ ________________________________**

Place, date Patient's signature

**___________________________ ________________________________**

Place, date Signature of the investigator
