# Supplementary material for: Yoga versus health education for persistent fatigue in patients with post-COVID-19 syndrome: protocol for a multicentre randomised controlled trial
Source: BMJ Open. 2025 Oct 23;15(10):e107840. doi: 10.1136/bmjopen-2025-107840 (PMC12551510; doi:10.1136/bmjopen-2025-107840)
Supplement: Supplementary file 2 [file bmjopen-2025-107840.reviewer_comments.pdf]

PEER REVIEW HISTORY

BMJ Open publishes all reviews undertaken for accepted manuscripts. Reviewers are asked to complete a checklist review form and are provided with free text boxes to elaborate on their assessment. These free text comments are reproduced below.

ARTICLE DETAILS

Title (Provisional)

Yoga versus health education for persistent fatigue in patients with post-COVID syndrome: protocol for a multicenter randomized controlled trial

Authors

Cramer, Holger; Moerchen, Lisa; Vagedes, Jan; Klenk, Jochen; Jäger, Simon; Schleinzer, Alina; Anheyer , Dennis; Bilc, Mirela; Schröter, Marleen; Kessler, Christian; Jeitler, Michael

---

VERSION 1 - REVIEW

**Reviewer**	**1**
**Name**	**Mumoli, Nicola**
**Affiliation**	**Department of Internal Medicine, Magenta Hospital, Italy**
**Date**	**14-Aug-2025**
**COI**	**none**

This planned randomized controlled trial comparing yoga to an active control (health education) is innovative, particularly given the high prevalence of post-COVID fatigue, the paucity of effective treatments, and the potential for yoga to offer both physical and psychological benefits. That said, several areas require further clarification or refinement:

1) The manuscript states the trial will include 100 participants, but the sample size calculation is not sufficiently detailed. The assumptions for effect size, variability, dropout rate, and the power analysis method should be clearly presented. Without these details, it is difficult to assess whether the study is adequately powered.

2) More detail is needed on how yoga instructors will be trained, certified, and monitored to ensure protocol adherence across centers.

The health education arm should also be described in equivalent detail to ensure comparability of attention and structure.

3) The authors note the risk of Post-Exertional Malaise (PEM) but provide limited detail on monitoring, criteria for detecting PEM, and predefined safety thresholds for modifying or discontinuing exercise.

Given the clinical significance, an explicit safety management protocol should be included.

4) While participant blinding is not possible, the protocol should specify whether outcome assessors (especially for performance tests and qualitative interviews) will be blinded to group allocation.

5) The statistical methods are described broadly but lack detail on handling missing data, adjustment for multiple comparisons, and sensitivity analyses. The plan for exploratory mediator/moderator analyses should clarify pre-specification to avoid data dredging concerns.

6) The economic evaluation is an important strength, but more specifics are needed:

- Which cost components will be included?

- How will productivity loss be measured?

- Will cost-utility analysis use QALYs derived from EQ-5D-5L?

7) The trial registration is included, but the relationship between protocol version 1.0 and the registered version should be explicitly stated to ensure transparency.

Minor Points:

Clarify if recruitment includes only patients meeting NICE post-COVID criteria or broader definitions.

Consider providing a schematic timeline of assessments and interventions for reader clarity.

Some sections (especially Background) could be slightly condensed to focus on rationale for the intervention and study design.

Reference formatting needs to be double-checked for consistency with journal guidelines.

VERSION 1 - AUTHOR RESPONSE

Thank you for your thoughtful and constructive feedback on our manuscript. We greatly appreciate the time and effort you have invested in reviewing our work. Please find our point-by-point reply attached, in which we have carefully addressed all your comments and suggestions.

We hope that our revisions meet your expectations and look forward to your feedback.

This planned randomized controlled trial comparing yoga to an active control (health education) is innovative, particularly given the high prevalence of post-COVID fatigue, the paucity of effective treatments, and the potential for yoga to offer both physical and psychological benefits. That said, several areas require further clarification or refinement:

1) The manuscript states the trial will include 100 participants, but the sample size calculation is not sufficiently detailed. The assumptions for effect size, variability, dropout rate, and the power analysis method should be clearly presented. Without these details, it is difficult to assess whether the study is adequately powered.

Response: **Thank you for this comment. We would like to note that details regarding the sample size calculation are included in the manuscript. The expected effect size was determined based on the minimal clinically relevant difference on the Chalder Fatigue Scale and population variability reported in the literature. The planned statistical analysis (two-tailed independent t-test) and the assumption of a 20% dropout rate, also based on published data, are described in the same section.**

2) More detail is needed on how yoga instructors will be trained, certified, and monitored to ensure protocol adherence across centers.

Response: We thank the reviewer for this valuable comment. We have now expanded the description of both intervention arms to provide more detail on instructor training, certification, and monitoring to ensure protocol adherence across centers. Specifically, we describe the certification of yoga teachers, the preparatory training they received, and the monitoring procedures implemented to ensure fidelity.

The health education arm should also be described in equivalent detail to ensure comparability of attention and structure.

Response: Likewise to the point above, we elaborated on the structure and delivery of the health education intervention to provide equivalent detail and ensure comparability in attention and group structure.

3) The authors note the risk of Post-Exertional Malaise (PEM) but provide limited detail on monitoring, criteria for detecting PEM, and predefined safety thresholds for modifying or discontinuing exercise.

Given the clinical significance, an explicit safety management protocol should be included.

Response: We thank the reviewer for raising this important point. We have now expanded the safety section of the manuscript to provide explicit detail on the monitoring and management of Post-Exertional Malaise (PEM). Adverse events (AEs), including PEM, are documented by participants on a weekly basis and reviewed by the study team every two weeks. Serious adverse events (SAEs) must be reported by participants immediately via direct phone contact with the study site. PEM is assessed using the validated PEM subscale of the DePaul Symptom Questionnaire (DSQ) for baseline and follow-ups, complemented by weekly symptom diaries. Safety thresholds for modifying or discontinuing exercise include (i) marked exacerbation of fatigue or other core symptoms persisting for >48 hours, (ii) deterioration in daily functioning, or (iii) occurrence of a SAE. Instructors and study physicians are authorized to adapt or interrupt the intervention in such cases. If participants are absent

from two sessions for health-related reasons, the site physician will discuss with them whether further participation remains safe and meaningful. We note that we did not apply a fixed cutoff score (e.g., Bell-Score) for exclusion; instead, participants' functional fitness and capacity were evaluated verbally in the physician-led informed consent visit, where potential risks and burdens of study participation were explained. This structured monitoring and predefined safety management protocol aims to minimize risks while allowing the inclusion of a representative post-COVID patient population.

4) While participant blinding is not possible, the protocol should specify whether outcome assessors (especially for performance tests and qualitative interviews) will be blinded to group allocation.

Response: We thank the reviewer for this important comment. While participant blinding is not feasible in behavioral interventions such as yoga or health education, outcome assessment will be conducted in a blinded manner wherever possible. Specifically, the study is designed as a single-blind randomized controlled trial, with outcome assessors blinded to group allocation. Performance tests (e.g., hand grip strength, physical activity sensors, and heart rate variability) are administered by study staff who are unaware of participants' group assignments. Likewise, laboratory analyses are performed in external laboratories without access to allocation data. For the qualitative interviews, the interviewers will not be involved in treatment delivery and will be instructed to avoid disclosure of group allocation during the interview process. Thus, although participants cannot be blinded, measures are in place to minimize the risk of bias by ensuring blinding of outcome assessors across both quantitative and qualitative components of the trial. We included these aspects in a new paragraph ("blinding").

5) The statistical methods are described broadly but lack detail on handling missing data, adjustment for multiple comparisons, and sensitivity analyses. The plan for exploratory mediator/moderator analyses should clarify pre-specification to avoid data dredging concerns.

Response: **Thank you for your comment. We would like to note that details regarding the handling of missing data, adjustment for multiple comparisons, and sensitivity analyses are included in the manuscript. Specifically, missing data will be imputed using a non-parametric approach with the missForest R package; no correction for multiple testing will be applied to secondary endpoints; and a per-protocol analysis will be performed as a sensitivity analysis.**

We agree that it is important to distinguish between pre-specified and exploratory analyses to address concerns about data dredging. In the manuscript, the planned mediation analyses focus on body awareness and self-efficacy as hypothesized mediators, while moderation analyses will examine the roles of personality traits and treatment credibility/expectations as potential moderators. We revised the manuscript to clarify this distinction and enhance transparency.

6) The economic evaluation is an important strength, but more specifics are needed:

- Which cost components will be included?

- How will productivity loss be measured?

- Will cost-utility analysis use QALYs derived from EQ-5D-5L?

Response: Thank you for your comment. We agree that the economic evaluation is a key strength of our study. We would like to note that details regarding cost categories, the measurement of productivity losses and the cost-utility analysis (including QALYs and EQ-5D-5L) are included in the manuscript (page 14).

- **Which cost components will be included?**
  Cost categories are classified according to Drummond's cost categories. The following cost categories will be included: interventions costs (e.g., instructor fees and equipment), direct medical costs (healthcare costs, e.g., outpatient care, inpatient care, and medication), non-medical costs (costs in other sectors, e.g., voluntary work; or patient and family costs, e.g., out-of-pocket expenses and informal care), and indirect costs (productivity losses). The study team will calculate intervention costs, while the other cost categories will be measured using a modified version of the CPAQ.
- **How will productivity loss be measured?**
  Participants were asked about their number of sick days, and any changes in their employment status during the intervention period This information will be used to calculate productivity losses using the human capital approach. Therefore, the productivity hours lost will be multiplied by the average national employment cost. Consequently, reduced work production due to sickness absence will be measured.
- **Will cost-utility analysis use QALYs derived from EQ-5D-5L?**
  Yes, the other economic evaluation studies on yoga that we identified used QALYs derived from the EQ-5D-5L. To enable comparison with these studies, and to adhere to the recommendations of the NICE guidelines in the UK and the IQWiG guidelines in Germany, we also plan to use QALYs derived from the EQ-5D-5L in our cost-utility analysis.

7) The trial registration is included, but the relationship between protocol version 1.0 and the registered version should be explicitly stated to ensure transparency.

Response: We thank the reviewer for this important comment regarding the relationship between the registered trial and the protocol versions. We have now clarified this aspect in the manuscript. The trial was initially registered on ClinicalTrials.gov (NCT05890599) on June 2, 2023, based on protocol version 1.0. All subsequent amendments (June 6, 2023, and June 14, 2025) and

updated protocol versions were communicated to ClinicalTrials.gov in a timely manner to ensure transparency. The current manuscript refers to protocol version 3.0 (January 17, 2025), which reflects these updates. This clarification has been explicitly added to the "Ethical approval, trial registration, and trial status" section of the manuscript.

Minor Points:

Clarify if recruitment includes only patients meeting NICE post-COVID criteria or broader definitions.

Response: We thank the reviewer for this important comment. Recruitment is based on the inclusion and exclusion criteria specified in the protocol. In line with the NICE guidelines, participants must have a confirmed SARS-CoV-2 infection (PCR or serology) and persisting symptoms lasting longer than twelve weeks, with self-reported fatigue attributed to COVID-19. Eligibility further requires a score ≥ 4 on the binomial Chalder Fatigue Scale. Thus, recruitment is not limited to the NICE definition alone, but operationalized through a combination of guideline-based symptom duration and validated fatigue measurement. This ensures that participants reflect both the NICE criteria for post-COVID condition and the clinically relevant persistence of fatigue.

Consider providing a schematic timeline of assessments and interventions for reader clarity.

Response: We thank the reviewer for this helpful suggestion. A schematic overview of assessments and interventions is already included in the manuscript (see Figure 1). This schedule of enrolment, interventions, and assessments provides a clear timeline for readers.

Some sections (especially Background) could be slightly condensed to focus on rationale for the intervention and study design.

Response: We thank the reviewer for this constructive comment. In line with the suggestion, we have condensed the Background section to reduce redundancy and place greater emphasis on the rationale for the intervention and the study design. The revised version highlights fatigue as the main symptom of post-COVID syndrome, its clinical relevance, and the rationale for yoga as a therapeutic approach.

Reference formatting needs to be double-checked for consistency with journal guidelines

Response: We have carefully reviewed and revised the reference formatting to ensure full consistency with the journal's guidelines.
